# Straightforward Generation of Ultrapure Off-the-Shelf Allogeneic CAR-T Cells

**DOI:** 10.3389/fbioe.2020.00678

**Published:** 2020-06-25

**Authors:** Alexandre Juillerat, Diane Tkach, Ming Yang, Alex Boyne, Julien Valton, Laurent Poirot, Philippe Duchateau

**Affiliations:** ^1^Cellectis Inc, New York, NY, United States; ^2^Cellectis, Paris, France

**Keywords:** chimeric antigen receptor (CAR) cells, adoptive immunotherapy, GvHD, cell purification, mRNA vectorization

## Abstract

Here, we developed a straightforward methodology to generate TCRαβ negative (allogeneic) cells for CAR-T cell therapies. With an early and transient expression of an anti-CD3 CAR in the engineered donor T cells, we programmed these cells to self-eliminate the TCR+ cell population and obtained an ultrapure TCRαβ^–^ population (99–99.9%) at the end of the CAR-T production. This novel and easy-to-implement procedure preserves the production yield and cell fitness and has the potential to streamline the manufacturing of “off-the-shelf” CAR T-cell therapies.

## Introduction

The adoptive transfer of engineered autologous T-cells has been a major breakthrough in the treatment of hematological cancer, leading to dramatic clinical remissions for patients with refractory B-cell leukemia (chronic lymphocytic leukemia and adult and pediatric acute lymphoblastic leukemia) and lymphoma (non-Hodgkin’s lymphoma) (Brudno and Kochenderfer, 2018; June and Sadelain, 2018). One attractive alternative to autologous T-cell transfer is the use of third-party donor-derived (allogeneic) T-cells, which would enable the generation of “off-the-shelf” universal CAR-T cells (UCAR T-cells) that are readily accessible to patients, reducing the cancer treatment decision to CAR T-therapy lag time intrinsic to individual autologous therapies. However, “off-the-shelf” cells derived from possibly non-HLA-matched donors carry the risk of graft-versus-host-disease through the expression of endogenous T-cell receptors (TCRs). To eliminate the expression of endogenous TCRs, genome editing approaches such as TALEN (Poirot et al., 2015; Valton et al., 2015; Qasim et al., 2017; Rasaiyaah et al., 2018), zinc fingers nucleases (Provasi et al., 2012), meganuclease (MacLeod et al., 2017), megaTAL (Hale et al., 2017), or CRISPR/Cas9 (Eyquem et al., 2017; Georgiadis et al., 2018) can be used to inactivate TCRαβ at the molecular level. However, these approaches require an additional column-based purification step to obtain an ultrapure TCRαβ^–^ population (>97%) at the end of the CAR-T production (Qasim et al., 2017). The therapeutic feasibility of using such engineered allogeneic CAR T-cells has been demonstrated by the molecular remission observed in patients after infusion of universal (allogeneic) TALEN multiplex gene-edited (CD52 and TCRαβ KO) CAR T-cells (Qasim et al., 2017).

**FIGURE 1:**
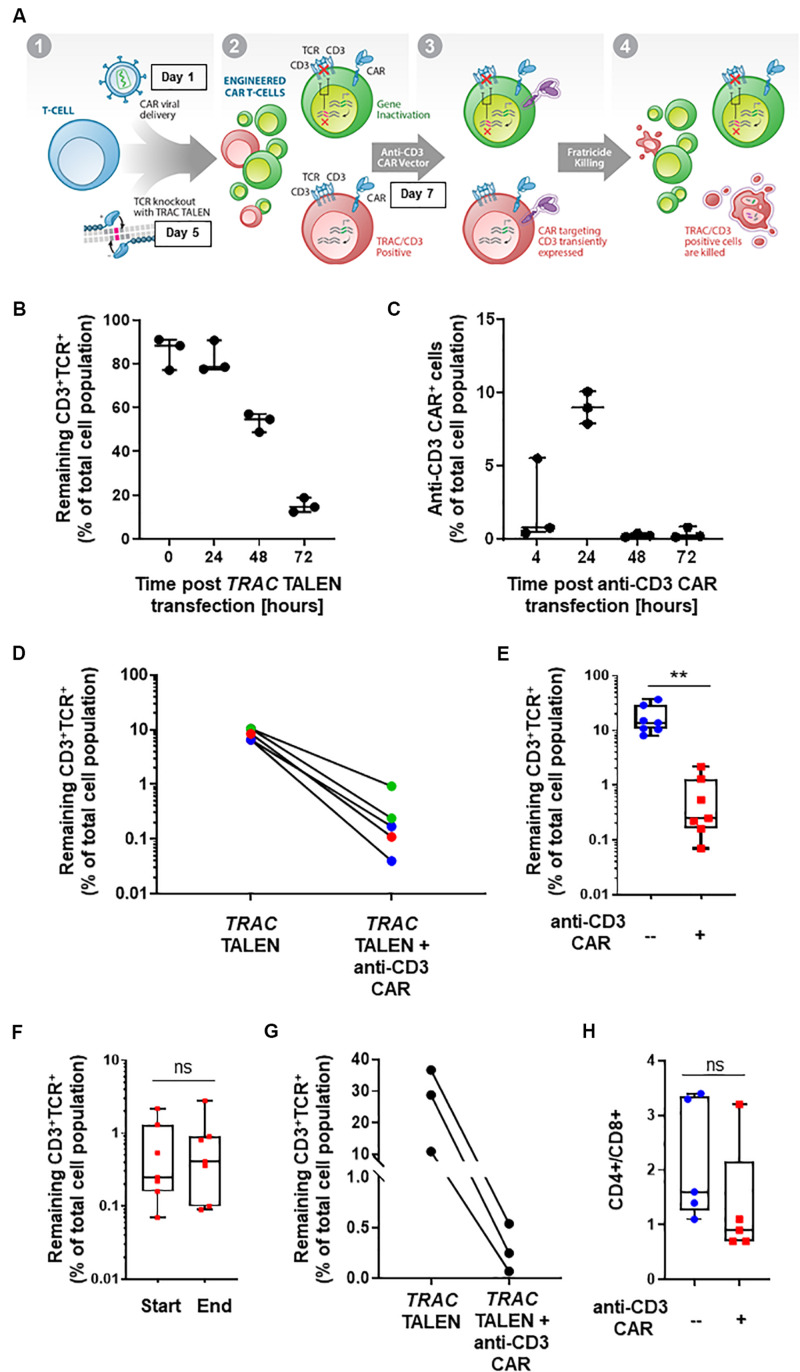
**(A)** Schematic representation of CD3+TCRαβ+ population elimination strategy. (1) T-cells are engineered to present a CAR via lentiviral viral delivery (day 1 post thawing) and to lack TCRα/CD3 via TALEN mRNA vectorization (day 5 post thawing). (2) A CAR targeting CD3 is transiently expressed via mRNA electroporation (day 7 post thawing). (3) Fratricide killing of the remaining TCRαβ/CD3+ CAR T-cells is induced upon anti-CD3 CAR expression. (4) Final UCAR *T*-cell product depleted (<1%) of TCRαβ/CD3+ cells. **(B)** Percentage of CD3^+^TCR^+^ cells remaining 24, 48, and 72 h post *TRAC* TALEN mRNA transfection (% of total cell population). *N* = 3, 3 independent T-cell donors. **(C)** Percentage of cells positive for the surface detection of the anti-CD3 CAR in *TRAC* KO T-cells at 4, 24, 48, and 72 h post mRNA transfection. *N* = 3, three independent *T*-cell donors. **(D)** Percentage of CD3+TCRαβ+ cells after consecutive transfections of *TRAC* TALEN mRNA and anti-CD3 CAR mRNA (in the absence of a CD22 CAR) spaced out by 2 (blue), 7 (green), and 9 days (red). **(E)** Percentage of CD3+TCRαβ+ cells after consecutive transfections of *TRAC* TALEN mRNA and anti-CD3 CAR mRNA 48 h apart. *N* = 7; 5 independent *T*-cell donors. **(F)** Remaining CD3+TCRαβ+ cells at the start (D11) and end (D17,18) of the expansion phase. *N* = 7, five independent T-cell donors. **(G)** Percentage of CD3+TCRαβ+ cells after consecutive transfections of *TRAC* TALEN mRNA and anti-CD3 CAR mRNA spaced out by 48 h; *T*-cell from donor 1, *N* = 1. **(H)** CD4+/CD8+ ratio of the *T*-cell population at the end of the expansion phase, with (red) or without (blue) transfection of the anti-CD3 CAR mRNA. *N* = 5; five independent *T*-cell donors. Significance is determined by a standard unpaired *t*-test, *p ≤ 0.05, **p ≤ 0.01.

Here, we aimed to develop a straightforward, column free, strategy to deplete remaining TCRαβ positive cells shortly after the gene editing step. To accomplish this approach, we sought to transiently leverage the killing properties of T-cells. In particular we anticipated that the transient expression, via mRNA electroporation, of a CAR targeting CD3 (Chen et al., 2016), a molecule associated with the TCRαβ complex at the cellular surface, in T-cells would result in the self-elimination of any remaining TCRαβ-positive cells (Figure 1A). Such an easy to implement, column free depletion approach would also limit the equipment requirement as the same device (electroporation) will be used for gene editing and autonomous purification of the engineered cells.

## Materials and Methods

### T-Cell Proliferation

Cryopreserved human PBMCs were acquired from ALLCELLS (cat #PB006F) and used in accordance with Cellectis IRB/IEC-approved protocols. T-cells were cultured in X-Vivo 15 medium (Lonza) supplemented with 5% human AB serum (Gemini) and 20 ng/ml IL-2 (Miltenyi) at a density of 1 × 10^6^ cells/ml.

### mRNA Production

mRNA was produced with EPAP-mediated polyadenylation using the mMessage mMachine T7 Ultra kit (Thermo Fisher Scientific) from a PCR product encoding the anti-CD3 CAR or without EPAP-mediated polyadenylation or from a linearized plasmid DNA template, encoding the anti-CD3 CAR, a mouse hba 3′UTR and a 120-nucleotide-long polyA.

### Lentiviral Particle Production

Lentiviral particles were generated in 293FT cells (Thermo Fisher Scientific) cultured in RPMI 1640 medium (Thermo Fisher Scientific) supplemented with 10% FBS (Gibco), 1% HEPES (Gibco), 1% L-Glutamine (Gibco) and 1% Penicillin/Streptomycin (Gibco) using Opti-MEM medium (Gibco) and Lipofectamine 2000 (Thermo Fisher Scientific) according to standard transfection procedures. Supernatants were recovered and concentrated by ultracentrifugation as indicated 48 and/or 72 h after transfection.

### T-Cell Transduction

Cryopreserved human PBMCs (ALLCELLS) were thawed and plated at a density of 1 × 10^6^ cells/ml in X-vivo-15 media (Lonza) supplemented with 5% human AB serum (Gemini) or CTS Immune Cell SR (Thermo Fisher Scientific) and 20 ng/ml IL-2 (Miltenyi Biotech) for an overnight culture at 37°C. The next day, the PBMCs were activated using human *T* activator CD3/CD28 (Life Technology) in serum-free X-vivo-15 media without IL-2. One million activated PBMCs (in 600 μl) were immediately incubated without removing the beads in an untreated 12-well plate pre-coated with 30 μg/ml Retronectin (Takara) in the presence of lentiviral particles encoding the CD22 targeting CAR for 2 h at 37°C. Six hundred microliters of 2× X-vivo-15 media (X-vivo-15, 10% human AB serum and 40 ng/ml IL-2) was added after 2 to 3 h, and the cells were incubated at 37°C for 72 h.

### T-Cell Transfection

Four days following activation/transduction, human *T* lymphocytes were transfected by electrotransfer using an AgilePulse MAX system (Harvard Apparatus). Cells were pelleted and resuspended in cytoporation medium T. 5 × 10^6^ cells were mixed with 5 μg total *TRAC* TALEN mRNA (2.5 μg each of the left and right TALEN arms) into a 0.4 cm cuvette. Separate aliquots of *TRAC* TALEN or mock-transfected cells were again electroporated at different time points (days 2, 7, or 9 post *TRAC* TALEN transfection) with 20 μg of anti-CD3 CAR mRNA. Engineered T-cells were then kept in culture for up to 4 days before expansion for 6,7 days in G-Rex10 (Wilson Wolf) in 40 ml of complete X-vivo-15 media.

### Flow Cytometry

The proportion of T-cells expressing the CAR or different markers at their surface was then quantified using the following antibodies: anti-CD3 CAR [according to (Chen et al., 2016)]: Biotin-labeled polyclonal goat anti-mouse F(Ab’)^2^ (Jackson Immunoresearch #115-065-07), streptavidin-APC (BD Bioscience #554067), CD3: Clone BW264/56, Vioblue (Miltenyi #130-094-363), TCRαβ: Clone REA652, PE (Miltenyi #130-109-920), CD4: Clone VIT4, PEVio770 (Miltenyi #130-096-552), CD8: Clone SK1, BV510 (Biolegend #344732), CD62L: Clone 145/15, APC (Miltenyi #130-113-617), CD45RA: Clone T6D11, Vioblue (Miltenyi #130-113-360), PD1: Clone REA1165, PE (Miltenyi #130-120-388) and LAG3: Clone 11C3C65, BV421 (Biolegend #369313).

### Antigen-Dependent Proliferation

Raji cells were treated with 20 Gy using a CellRad X-ray irradiation system (Faxitron), washed twice, and counted. A total of 500,000 Raji cells were plated with 500,000 T-cells (1:1) in duplicate into 1 ml final volume of X-vivo-15 medium with 5% human AB serum, but lacking IL-2, in a 24 well plate. At days 4 and 7, the cells were counted on a Vi-Cell Cell Counter (Beckman Coulter) and passaged at 500,000 cells/0.5 ml media into a 48-well plate. At day 10, the cells were mixed and counted for the last time point.

### Assessment of CAR Cytotoxicity

Transduced T-cells (1.5 × 10^6^ cells) were incubated in X-vivo-15 medium with 5% human AB serum, lacking IL-2 in a 3:1 (T-cells) ratio to target cells (Raji) presenting the CAR target antigen and expressing a luciferase (0.5 × 10^6^ cells) in a 12-well plate. After 24 h, the cells were collected, and 100 μl of cells was used for luciferase quantification (ONE-Glo, Promega). The remaining cells were pelleted and resuspended in fresh X-vivo 15 media with 5% human AB serum, no IL-2, and an additional 0.5 × 10^6^ target cells were added. This step was repeated for two consecutive days.

## Results and Discussion

With the goal of developing a CAR-based TCRαβ^+^ cell depletion strategy, we focused on a second-generation CAR construct containing a single-chain variable fragment (scFv) targeting the CD3 antigen. We used a hinge and transmembrane domains derived from the T-cell surface glycoprotein CD8 alpha chain (CD8a) and intracellular signaling domains from co-stimulatory 4-1BB (CD137) and activation of the ζ-chain of the CD3–T cell receptor. First, we evaluated the timing of the *TRAC* TALEN and anti-CD3 CAR mRNA transfections that would allow a reduction in TCRα/CD3 surface presentation prior to the anti-CD3CAR surface expression. We therefore monitored the kinetic of inactivation of the *TRAC* gene, as reported by the decrease of CD3 and TCRαβ surface expression without transfection of the aanti-CD3 CAR (Figure 1B). We observed a strong reduction of CD3 and TCRαβ surface expression at 48h and 72h post *TRAC* TALEN mRNA transfection (1.6- and 5.3-decrease, respectively, Figure 1B). We then looked at the surface expression of the anti-CD3 CAR in *TRAC* KO T-cells following the anti-CD3 CAR mRNA transfection (Figure 1C). We found that the anti-CD3 CAR was expressed in ∼10% of the cell population 24 h post mRNA transfection while expression has completely ceased at 48 h (Figure 1C). We next sought to monitor the effects of transiently expressing the anti-CD3 CAR (mRNA transfection) at different timing after TALEN-based TCRαβ inactivation of peripheral T-cells as early transfection will facilitate large-scale manufacturing with less cells to be electroporated (Poirot et al., 2015). Independent of the anti-CD3 CAR transfection time point (2, 7, or 9 days after TALEN treatment), we observed a significant elimination of the remaining CD3+TCRαβ+ population, from 6.5–10.4% positive cells to 0.04–0.93% (median 0.17%) in the anti-CD3 CAR mRNA-treated samples, corresponding to an overall 91–99% depletion efficiency (median, 97.7%; Figure 1D). We next sought to determine whether the depletion capacity of transiently expressed anti-CD3 CAR was impaired in cells that were otherwise stably expressing another CAR targeting a potential tumor antigen (CD22) through lentiviral vectorization (Xiao et al., 2009). We developed a protocol, starting from frozen PBMCs, which incorporated a lentiviral particle transduction step (CD22 CAR integration) and two mRNA transfection steps. We carried out a first mRNA electroporation (*TRAC* TALEN), followed 48 h later by the anti-CD3 CAR electroporation. Four days post anti-CD3 CAR electroporation, we observed a minimal residual CD3+ TCRαβ+ population (median CD3+ TCRαβ+: 0.25%, median depletion: 98.5%), indicating that the prior presence of a stably expressed antitumor CAR does not affect the CD3 depletion ability of the cells (*p*-value: 0.0013, Figure 1E). These engineered UCAR-T cell populations were then further expanded for 10 to 11 days in the presence of IL-2. We did not observe any expansion bias due to the anti-CD3 CAR transient expression on the total cells collected at the end of the process (*p*-value: 0.969, Supplementary Figure 1), with the CD3+ TCRαβ+ population remaining unaffected (*p*-value: 0.8252, Figure 1D) and minimal (median: 0.41%, Supplementary Figure 2). The fact that the CD3+ TCRαβ+ population remained unaffected between the beginning and end of the expansion process is pointing toward an absence of long term anti-CD3 CAR driven killing. Overall, we obtained a cell population that contains less than 1% of residual TCRαβ T-cells for four out of five donors tested (Figure 1E). Furthermore, we monitored depletion in function of the starting percentage of CD3 positive cells. We observed efficient elimination of CD3+ TCRαβ+ (98.7–99.6% depletion) within a given T-cell donor, independently of the starting percentage of CD3 positive cells obtained after gene editing (in the range of 10–36%, Figure 1G). The possibility to deplete samples presenting a relatively high starting CD3+ TCRαβ+ population represents a first step toward a future translation to clinically relevant conditions.

**FIGURE 2:**
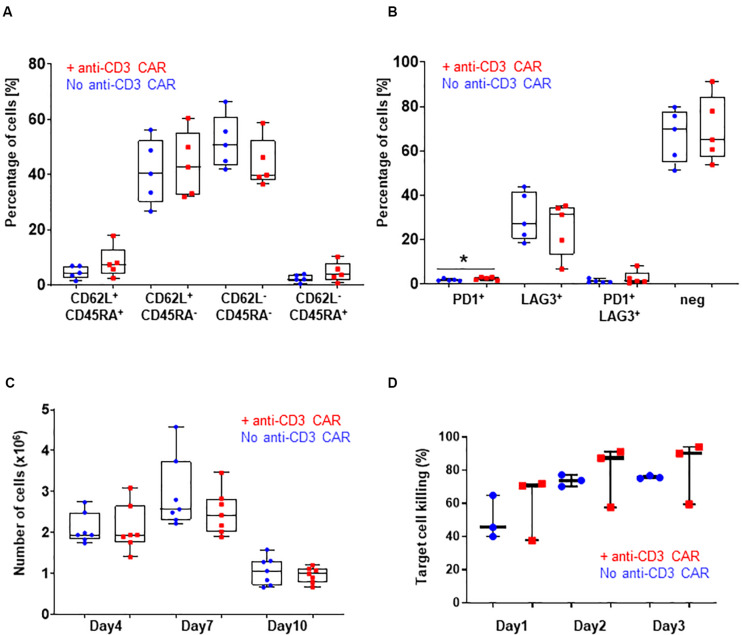
**(A)** CD62L/CD45RA expression in the CD8 population with (red) or without (blue) transfection of the anti-CD3 CAR mRNA. N=5; 5 independent T-cell donors. p-value: 0.2251 for CD45RA^+^ CD62L^+^, 0.7472 for CD45RA^+^ CD62L^–^, 0.2185 for CD45RA-CD62L^–^, and 0.2327 for CD45RA-CD62L^+^ : **(B)** PD1 and LAG3 expression in the whole T-cell population with (red) or without (blue) transfection of the anti-CD3 CAR mRNA. N=5, 5 independent T-cell donors p-value: 0.1307 for PD1, 0.5295 for LAG3, 0.3597 for PD1/LAG3 and 0.7521 for double negatives. **(C)** Antigen-dependent proliferation of CAR T-cells with (red) or without (blue) transfection of the anti-CD3 CAR mRNA over a 10-day period. N=7; five independent T-cell donors. **(D)** Target cell killing over a period of 3 days. UCAR T-cells engineered with (red) or without (blue) transfection of the anti-CD3 CAR mRNA. N=3; three independent T-cell donors. Significance is determined by a standard unpaired t-test, *p ≤ 0.05, **p ≤ 0.01.

Knowing that the expression, even transient, of a CAR could impact the fitness of CAR T-cells, we further analyzed three characteristics of the T-cell populations generated from the five individual donors: (i) the CD4-to-CD8 ratio, (ii) T-cell differentiation, and (iii) exhaustion markers. The samples transfected with the anti-CD3 CAR showed a moderate CD8^+^-biased skewing unlike untransfected samples (with the exception of one donor that failed to reach <1% of residual TCRαβ after treatment), overall maintaining a balanced CD4^+^ to CD8^+^ ratio (*p*-value: 0.2549, Figure 1H). The proportion of terminal effector cells, which were associated with reduced *in vivo* anti-tumor functions (CD45RA^+^CD62L^–^) (Gattinoni et al., 2011; Sommermeyer et al., 2016), was not significantly affected by the anti-CD3 CAR (*p*-value: 0.7472, Figure 2A and Supplementary Figure 3). In addition, both anti-CD3 CAR-transfected and untransfected T-cells contained large proportions of effector memory (CD45RA^–^CD62L^+^) and naïve/central memory cells (CD62L^+^) cells, which are associated with improved anti-tumor function (Figure 2A and Supplementary Figure 3) (Gattinoni et al., 2011; Sommermeyer et al., 2016). Because the acquisition of an exhausted phenotype has been previously associated with reduced *in vivo* functions, we monitored the surface expression of PD1 and LAG3, two well documented exhaustion markers (Blackburn et al., 2009). Overall, we observed low frequencies of PD1, LAG3, or PD1/LAG3 co-expressing cells in the anti-CD3 CAR-treated samples (Figure 2B), without substantial upregulation when compared to the untreated samples.

We then closely examined the fitness and function of the engineered UCAR T-cells *in vitro* and *in vivo*. We first monitored T-cell proliferation over a period of 10 days in response to a single stimulation with target cells expressing the appropriate antigen (CD22+, Raji) in the absence of IL2. This experimental setup did not reveal marked differences in the antigen-dependent proliferative capacity of the UCAR T-cells treated or not with the anti-CD3 CAR (standard unpaired *t*-test: *p*-values: 0.9957 for day 4, 0.2596 for day 7 and 0.5015 for day 10, Figure 2C). Using engineered UCAR T-cells from three donors, we performed an *in vitro* three days killing assay where the UCAR T-cells were challenged with an additional target cell load every day (luciferase expressing Raji cells). In this stringent *in vitro* model, the UCAR T-cells engineered with or without anti-CD3 CAR treatment promoted target cell killing at similar efficiency, as measured by target cell luciferase signal (standard unpaired t-test: p-values: 0.5053 for day1, 0.6696 for day 2 and, 0.6500 for day 3, Figure 2D).

Although extensive *in vivo* testing will be required to fully assess the potential of this technology before clinical applications, this study provides a proof of concept methodology to produce the next generation of “off-the-shelf” CAR T-cells. Upon future demonstration of scale-up validation using clinically relevant conditions and GMP material, we believe that this method has the potential to streamline manufacturing of “off-the-shelf” CAR T-cell therapies. In summary, we propose a novel, modular and broadly implementable methodology that can efficiently eliminate residual TCRαβ+ cells during the early steps of the allogeneic CAR T-cell generation process, with the aim to reduce the risk of, or even prevent, graft-versus-host-disease, without altering key characteristics (T-cell differentiation, exhaustion markers, proliferative capacity and target cell killing capacity) of these allogenic engineered UCART cells.

## Data Availability Statement

The datasets generated for this study are available on request to the corresponding author.

## Author Contributions

AJ, MY, AB, LP, and PD conceived the study and designed the experiments. AJ, MY, and DT performed the experiments. JV provided the conceptual advice and technical support. AJ, DT, and LP analyzed the experiments. AJ, LP, and PD wrote the manuscript with support from all authors. All authors contributed to the article and approved the submitted version.

## Conflict of Interest

AJ, DT, MY, AB, JV, LP, and PD are employed by the company Cellectis.
