# Supplementary material for: Straightforward Generation of Ultrapure Off-the-Shelf Allogeneic CAR-T Cells
Source: Front Bioeng Biotechnol. 2020 Jun 25;8:678. doi: 10.3389/fbioe.2020.00678 (PMC7330105; doi:10.3389/fbioe.2020.00678)
Supplement: Supplementary file 1 [file Image_1.pdf]

Supplementary information for:

Straightforward generation of ultrapure off-the-shelf allogeneic CAR-T cells

Alexandre Juillerat[1], Diane Tkach[1], Ming Yang[1], Alex Boyne[1], Julien Valton[1], Laurent Poirot[2] and Philippe Duchateau[2]

[1]Cellectis Inc, 430E, 29th street, NYC, NY 10016, USA

[2]Cellectis, 8 rue de la croix Jarry, 75013 Paris

Corresponding authors: alexandre.juillerat@cellectis.com and philippe.duchateau@cellectis.com

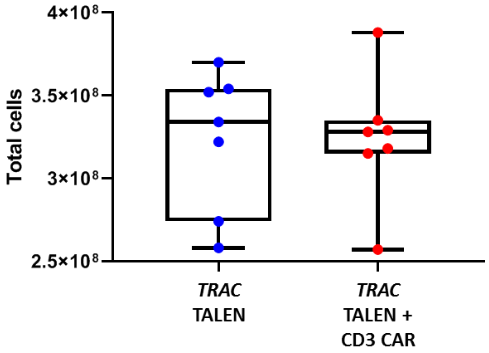

Supplementary Figure 1. Total number of cells collected at the end of the expansion phase.

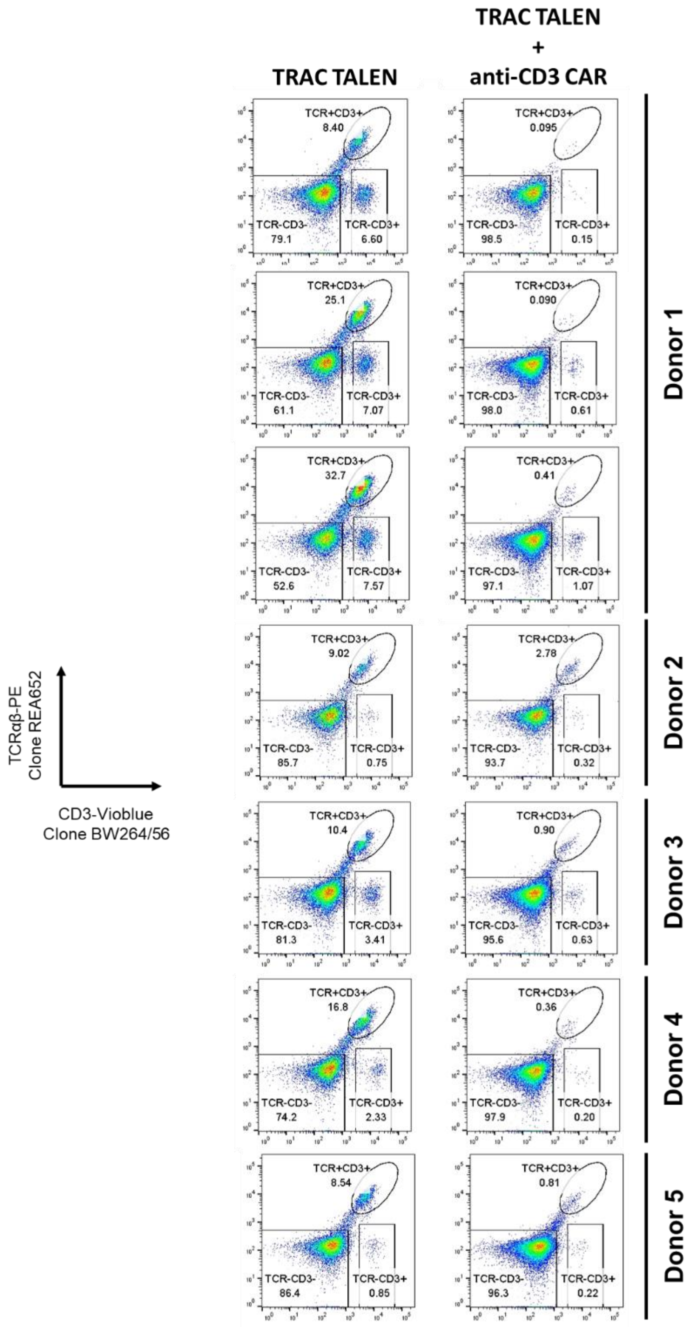

Supplementary Figure 2. Flow cytometry dot plots of CD3 and TCRαβ surface detection at the end of the expansion process after freezing and thawing.

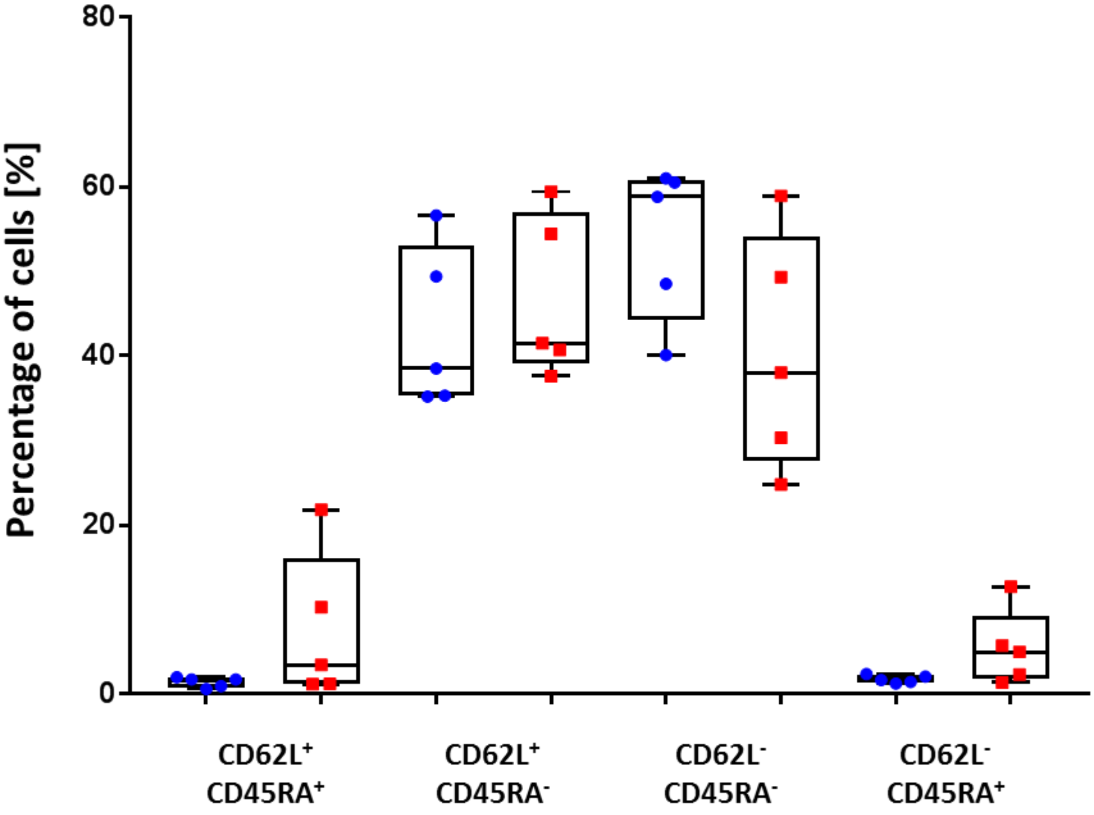

Supplementary Figure 3. CD62L/CD45RA expression in the CD4+ population with (red) or without (blue) transfection of the anti-CD3 CAR mRNA. N=5; 5 independent T-cell donors.
